# Dietary (−)-epicatechin as a potent inhibitor of βγ-secretase amyloid precursor protein processing^☆^

**DOI:** 10.1016/j.neurobiolaging.2014.07.032

**Published:** 2015-01

**Authors:** Carla J. Cox, Fahd Choudhry, Eleanor Peacey, Michael S. Perkinton, Jill C. Richardson, David R. Howlett, Stefan F. Lichtenthaler, Paul T. Francis, Robert J. Williams

**Affiliations:** a Department of Biology and Biochemistry, University of Bath, UK; b Wolfson Centre for Age-Related Diseases, King's College London, London, UK; c Neurosciences Therapy Area Unit, GlaxoSmithKline, Medicines Research Centre, Hertfordshire, UK; d Neuroproteomics, Klinikum rechts der Isar, Technische Universität München, Munich, Germany; e German Center for Neurodegenerative Diseases (DZNE), Munich, Germany; f Munich Cluster for Systems Neurology (SyNergy), Munich, Germany

**Keywords:** Flavanol, Flavonoid, Alzheimer's disease, TASTPM, BACE1, Amyloid pathology, Aβ, Dietary polyphenolic, Dementia, APP processing, Catechin

## Abstract

Flavonoids, a group of dietary polyphenols have been shown to possess cognitive health benefits. Epidemiologic evidence suggests that they could play a role in risk reduction in dementia. Amyloid precursor protein processing and the subsequent generation of amyloid beta (Aβ) are central to the pathogenesis of Alzheimer's disease, as soluble, oligomeric Aβ is thought to be the toxic species driving disease progression. We undertook an in vitro screen to identify flavonoids with bioactivity at βγ-mediated amyloid precursor protein processing, which lead to identification of a number of flavonoids bioactive at 100 nM. Because of known bioavailability, we investigated the catechin family further and identified epigallocatechin and (−)-epicatechin as potent (nanomolar) inhibitors of amyloidogenic processing. Supporting this finding, we have shown reduced Aβ pathology and Aβ levels following short term, a 21-day oral delivery of (−)-epicatechin in 7-month-old TASTPM mice. Further, in vitro mechanistic studies suggest this is likely because of indirect BACE1 inhibition. Taken together, our results suggest that orally delivered (−)-epicatechin may be a potential prophylactic for Alzheimer's disease.

## 1 Introduction

Since its conception in the early 1990s, the amyloid cascade hypothesis has remained central to research into the development of therapeutics for the treatment of Alzheimer's disease (AD) (Hardy and Higgins, 1992). It states that misregulation of amyloid beta (Aβ) production leads to Aβ accumulation and deposition, aberrant signaling, and downstream neurotoxicity (Hardy, 2009, Hardy and Allsop, 1991, Hardy and Higgins, 1992). The lack of successful drugs targeting Aβ, however, has created a significant unmet need for alternative approaches to AD treatment.

Flavonoids are a group of several thousand polyphenolic compounds distinguished by the presence of 2 phenyl rings connected by benzene. They are subcategorized into 6 groups dependent on structure: flavanols, flavanones, flavonols, flavones, isoflavones, and anthocyanins. As secondary metabolites in fruits and vegetables, flavonoids are present in human diet (Commenges et al., 2000, Letenneur et al., 2007). Once ingested, flavonoids are extensively metabolized; they can be glycosylated, methoxylated, sulfated, esterified, or polymerized (Manach et al., 2004). There is great variability in flavonoid bioavailability; many are directly excreted but some flavonoids or their metabolites are detectable in the bloodstream hours after ingestion (Ramassamy, 2006). Although difficult to analyze because of extensive metabolism, it has been shown that concentrations of approximately 200–400 nM of flavonoid can be achieved in the brain (Abd El Mohsen et al., 2002, Wang et al., 2012).

The actions of flavonoids have classically been attributed to their antioxidant properties (Rice-Evans et al., 1996). This has recently been questioned because of low concentrations of flavonoids achievable in the brain, meaning they are unlikely to be beneficial over the endogenous antioxidants such as ascorbate (Williams et al., 2004). Transgenic mouse models of AD have been used to demonstrate that selected flavonoids have beneficial effects on different aspects of the pathology. Tg2576 mice orally administered the flavanol epigallocatechin gallate (EGCG), showed reduced amyloid pathology, and improved cognitive function (Rezai-Zadeh et al., 2008); this was shown in a separate study to be via activation of ADAM10, the α-secretase (Obregon et al., 2006). A flavonoid-rich grape seed polyphenolic extract orally delivered for 5 months also showed reduced Aβ pathology; however, this was shown to be via inhibition of Aβ aggregation (Wang et al., 2008). Monomeric synthetic 3′-O-methyl-epicatechin-5-O-glucuronide derived from the grape seed polyphenolic extract, when given orally for 5 months, showed positive effects on synaptic transmission and long-term potentiation, through the CREB signaling (Wang et al., 2012). Although these studies show promising results for flavonoids, they have typically been carried out on a chosen compound or small subset of compounds, looking at their effects in isolation and there have been inconsistencies in findings and lack of a coherent, systematic approach to looking across the family as a whole.

The challenge is to study the actions of flavonoids so that the most effective molecules for the modulation of amyloid precursor protein (APP) processing can be identified. We systematically assessed a range of isolated flavonoid compounds from across the different groups for inhibition of βγ-secretase mediated APP processing using an established βγ reporter assay. From this initial in vitro screen, the flavanol subfamily were focused on for more detailed concentration and kinetic analyses followed by in vivo analysis of the most potent inhibitory compound, (−)-epicatechin. Oral (−)-epicatechin reduced Aβ pathology in TASTPM mice providing further evidence for the development of monomeric flavonoid compounds as therapeutic agents for the treatment or prevention of AD.

## 2 Methods

### 2.1 Compounds

Flavonoids were purchased from Extrasynthese and made up as 10 mM stocks in methanol. N-[n-(3,5-difluorophenactetyl)-L-alanyl]-(S)-phenylglycine t-butyl ester (DAPT) was purchased from Tocris. TAPI-1 was purchased from Enzo Life Sciences. APP β-secretase inhibitor (βsI) and β-secretase inhibitor IV (βIV) were purchased from Calbiochem. TAPI, DAPT, βsI, and βIV were made up as 1000× concentrated stocks in DMSO (Sigma).

### 2.2 Plasmids

pRC-CMV-APP_695_-Gal4 and pRC-CMV-APP_695_-Gal4Δ were as described previously (Hoey et al., 2009). pFR-Luciferase reporter vector with firefly (*Photinus pyralis*) luciferase gene under the control of a synthetic promoter containing 5 tandem repeats of the yeast Gal4 upstream activation sequence upstream of a minimal TATA box and phRL thymidine kinase (TK) vector containing sea pansy (*Renilla reniformis*) luciferase gene under control of the herpes simplex virus–TK promoter, from Promega. pC1-CMV vector containing complementary DNA (cDNA) encoding human Fe65 has been described previously (Perkinton et al., 2004). pSecTag2 vector containing cDNA encoding for human Notch3 fused in frame at its C terminus to the yeast transcription factor GAL4 was kindly provided by Dr Michael Perkinton (King's College, London).

### 2.3 pRC-APP_695_-Gal4 site-directed mutagenesis

pRC-CMV vector containing a cDNA encoding for the Swedish familial mutation of human APP695 fused in-frame at its C-terminus to the Gal4 yeast transcription factor was created using pRC-APP_695_-Gal4 plasmid (kind gift of Prof. Tommaso Russo, University of Naples) and introduction of a double point mutation at positions 595/596 of APP_695_ using the QuikChange XL Site-Directed Mutagenesis kit according to the manufacturer's instructions (Stratagene). Mutagenesis primers used APPswe forward primer 5′-GAGATCTCTGAAGTGAATCTGGATGCAGAATTCCGA-3′ and APPswe reverse primer 5′-TCGGAATTCTGCATCCAGATTCACTTCAGAGATCTC-3′.

### 2.4 Primary neuronal culture

Primary cortical neuronal cultures were prepared as described previously (Hoey et al., 2009). Cortices were dissected from embryonic day 15 CD1 or TASTPM embryos and mechanically dissociated using a serum-coated fire-polished Pasteur pipette in PBS + 6 mM D-glucose (Ca^2+^ and Mg^2+^ free). Neurons were plated into multiwell tissue culture plates (Nunc) that were precoated with 20 μg/mL poly-D-lysine (Sigma) and were maintained in Neurobasal medium minus phenol red, supplemented with B-27, 2 mM glutamine, 100 μg/mL streptomycin, and 50 μg/mL penicillin (Invitrogen), at 37 ^°^C in a humidified atmosphere of 95% air and 5% CO_2_. Cultures were used after 5–10 DIV.

### 2.5 Dual-Glo luciferase gene reporter assay

pRC-APP_695_-Gal4, pFR-Luciferase, pRC-APP_695_-Gal4DBD, pSecTag2-Notch3Gal4, and pC1-Fe65 plasmids (all 0.5 μg) were transfected into primary cortical neurons cultured in 12-well plates (0.5 × 10^6^ cells per well) at 5 DIV, using Lipofectamine 2000 (1 μL/well), as detailed in legend. All wells were cotransfected with phRL-TK Renilla (0.5 μg) as in internal control for luciferase expression. Transfection mixes containing lipid and DNA were prepared separately in OptiMEM I reduced serum medium (Invitrogen), then mixed and incubated at RT for 25 minutes. Neuronal cultures were removed from the incubator and 150 μL/well transfection mix was added dropwise to the culture medium. Cultures were then placed back in the incubator. Transfected neurons were treated with different compounds as detailed in legends and processed 24–30 hours posttransfection for quantification of firefly luciferase and Renilla luciferase expression. Neurons were lysed with Glo lysis buffer (100 μL/well) (Promega), and the Dual-Glo luciferase activity assay was performed according to the manufacturer's instructions (Promega). Luciferase signals were captured using a microplate luminometer (BMG Labtech). Firefly luciferase reporters activity was normalized using the Renilla luciferase activity, which helps to differentiate between specific and nonspecific cellular responses and control for transfection efficiencies across experiments.

### 2.6 Recombinant BACE1 assay

The BACE1 activity detection assay was performed according to the manufacturer's instructions (Sigma, MO, USA). Assay components were mixed to a final volume of 100 μL in a black 96-well microplate. Test samples were incubated with inhibitors or flavonoid compounds as indicated in the figure legends. Fluorescence readings (excitation 320 nm and emission 405 nm) were taken at time zero and 2 hours, and the fluorescence levels were measured. Stop solution was then added.

To measure endogenous BACE1 activity levels in 6 DIV primary cortical cultures (3.3 × 10^5^ cells per well) were treated, as detailed in figure legends, directly into the conditioned media. Following treatment, the media were aspirated, and wells were washed once with cold PBS. CelLytic M (200 μL; Sigma, MO, USA) was added to each well. Wells were then scraped and cellular material transferred to fresh microcentrifuge tubes. These were then centrifuged (2000× *g* for 5 minutes) and the supernatant transferred to a new microcentrifuge tube. Cell lysates (30 μL) were then incubated with synthetic BACE1 substrate, and fluorescent levels were measured as per the cell free assay protocol.

### 2.7 Detection of APP695 and APP C-terminal fragments

Primary cortical neurons cultured in 6-well plates (2 × 10^6^ cells per well) for 7–10 DIV were treated (as detailed in legends), washed once with cold PBS, and lysed in SDS-PAGE sample buffer (62.5 mM Tris, pH 6.8, 2% SDS, 5% 2-mercaptoethanol, 10% glycerol, and 0.0025% bromophenol blue), centrifuged, and then boiled for 5 minutes. Samples were resolved by 8% Tris-glycine SDS-PAGE for APP695 and by 16.5% Tris-tricine SDS-PAGE for APP C-terminal fragments (CTFs) (Hoey et al., 2009). After gel electrophoresis, proteins were transferred to 0.45 μm nitrocellulose membrane (GE Healthcare) for APP695 and to 0.2 μm PVDF (Millipore) for APP CTFs. Immunoblotting for APP695 was performed using primary antibody for total APP-22C11 (1:2000, Chemicon) and HRP-conjugated goat anti-rabbit IgG secondary Ab (1:5000, Millipore). Immunoblotting for β tubulin was performed using β tubulin primary monoclonal β tubulin Ab MAB380 – (1:10,000; Millipore) and HRP-conjugated goat anti-mouse IgG secondary Ab (1:5000; dilution Millipore). Immunoblotting for APP CTFs was performed using primary polyclonal APP Abs CT20 (raised to the final 20 amino acids in the C-terminal of APP695 (1:75,000) and custom synthesized by Eurogentech, Southampton, UK; (Perkinton et al., 2004) and HRP-conjugated goat anti-rabbit IgG secondary Ab (1:200,000; Millipore). Immunoblotting for sAPPα was performed using 5G11 (rat monoclonal) antibody supernatant (1:9) courtesy of SFL (Colombo et al., 2012) and HRP-conjugated goat anti-rat IgG secondary Ab (1:5000, Millipore). APP695, sAPPα, and β tubulin were detected using the ECL system (GE Healthcare) and APP CTFs by ECL Advance system (GE Healthcare), followed by exposure to Hyperfilm ECL according to the manufacturer's instructions (GE Healthcare).

### 2.8 (−)-Epicatechin feeding study

All animal studies were ethically reviewed and carried out in accordance with Animals (Scientific Procedures) Act 1986 and the GSK Policy on the Care, Welfare, and Treatment of Animals. TASTPM mice (male), 7 months old, were housed individually in standard cages. Mice were randomly assigned to groups and drank (−)-epicatechin supplemented water (3 mg/mL) or vehicle (0.1% ethanol vol/vol) for 21 days in 5 mL sipper tubes so that liquid intake could be measured. Intake was estimated to be close to 15 mg (−)-epicatechin a day, shown previously to equate to nanomolar brain concentration (Wang et al., 2012). At the end of the experiments, animals were sacrificed and the brains rapidly removed and hemisected. One hemisect was immediately immersion fixed in 4% paraformaldehyde in 0.1 M phosphate buffer saline (PBS, pH 7.4) for 48 hours and used subsequently for immunohistochemistry. The cortex was dissected from the remaining hemisect and frozen on dry ice to be used for enzyme-linked immunosorbent assay (ELISA).

### 2.9 Immunohistochemistry

Immunohistochemistry for Aβ plaques was performed on 5 μm sagittal sections. The antibody used was 20G10 (Glaxo Smith Kline, Harlow, UK; 0.28 μg/mL) (Howlett et al., 2004) raised against the Aβ_35–42_ fragment and selected for its C-terminal Aβ_42_ specificity. Immunohistochemistry was completed with appropriate secondary biotinylated antibodies (Vector Laboratories, Peterborough, UK) diluted in 1:500 in secondary layer diluent (0.3% Triton-X-100 in 0.1 M PBS), followed by avidin-biotin complexation (Vector ABC, Vector Laboratories), and visualized using diaminobenzidine according to the manufacturer's data sheets (Vector Laboratories). The percentage of the total section area labeled by the Aβ antibody in representative sections from each mouse fed either the control or (−)-epicatechin diet was determined using Qwin software macros (Leica, V2.0) (Howlett et al., 2004).

### 2.10 Measurement of Aβ from TASTPM cultures

TASTPM cortical cultures (7 DIV) were treated with either (Qwin software)-epicatechin (100 nM), DAPT (10 μM), or vehicle for 6 hours and human Aβ_1-40_ levels in the media measured by ELISA using a mouse monoclonal capture antibody 1A10 (IBL, Hamburg) which picks up residues 35-40 of human Aβ_1-40_.

### 2.11 Measurement of Aβ from brain homogenates

A Bio Veris immunoassay was used to measure the levels of Aβ_1-40_ and Aβ_1-42_ in formic acid extracted cortical homogenates from (−)-epicatechin and vehicle supplemented TASTPM mice. 6E10 antibody (Senetek, Maryland Heights, MO, USA) was used to capture the entire Aβ molecule and Bio Veris tagged G210 or 5G5 were used to detect Aβ_1-40_ and Aβ_1-42_, respectively.

### 2.12 Quantification and statistics

Mean data ± standard error of the mean were graphed using GraphPad Prism 6 software. Immunoblot, Aβ_x-40_ ELISA, and Dual-Glo luciferase activity assay data were analyzed by 1-way analysis of variance with Bonferroni posttest or by 2-tailed Student t test, using GraphPad Instat software. Differences between treatments were defined as statistically significant when *p* < 0.05.

## 3 Results

### 3.1 APP-Gal4 driven luciferase gene reporter activity preferentially reports amyloidogenic processing in primary cortical neurons

**Fig. 1:**
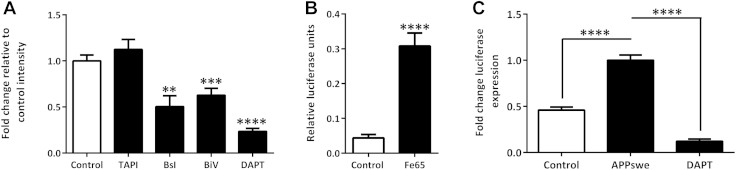
APP-Gal4 driven luciferase gene reporter activity preferentially reports amyloidogenic processing in primary cortical neurons. Five DIV primary mouse cortical neurons chemically transfected with 0.5 μg pRC-APP-Gal4, pFR-luciferase, and pRL-TK-Renilla plasmids using 1 μL/well Lipofectamine-2k. (A) Differential effects of alpha, beta, and gamma secretase inhibitors on APP-Gal4 gene reporter indicate assay is sensitive to amyloidogenic processing. ** *p* < 0.01, *** *p* < 0.001, and **** *p* < 0.0001 1-way ANOVA with Bonferroni posttest. (B) Promotion of luciferase signal following cotransfection with 0.5 μg adaptor protein Fe65. **** *p* < 0.0001 Student t test. (C) Cotransfection with APP-Gal4 plasmid point mutated at “Swedish” K595N/M596L (APP695 numbering) compared with WT transfection caused increase in processing, sensitive to 10 μM DAPT. **** *p* < 0.0001 1- way ANOVA with Bonferroni posttest. Abbreviations: ANOVA, analysis of variance; APP, amyloid precursor protein.

A cell based reporter gene assay has been established in primary cortical neurons, which allows identification of γ-secretase mediated APP processing modulators (Hoey et al., 2009). This approach has been used to screen a library of dietary flavonoids. As it has been proposed that the amyloidogenic and non-amyloidogenic pathways exist in equilibrium (Colombo et al., 2012, Suh et al., 2013) and rodent primary neurons appear to favor a BACE1 processing route (Hoey et al., 2013), it was first necessary to determine the relative contributions of the α-, β-, and γ-secretase enzyme activities. Treatment with the γ-secretase inhibitor DAPT reduced luciferase expression by approximately 80% (Fig. 1A), indicating that most luciferase expression is driven by γ-secretase-mediated APP cleavage. DAPT did not affect Renilla expression or induce any morphologic changes in the neurons suggesting that this reduction in luciferase expression was not because of toxicity. APP undergoes regulated intramembrane proteolysis; therefore, cleavage by the α- or β-secretase must occur before γ-secretase cleavage. Treatment with 2 structurally distinct BACE1 inhibitors, βsI, and βIV caused approximately 50% reduction in luciferase expression (Fig. 1A). TAPI, a broad-spectrum metalloprotease inhibitor which reduces α-secretase activity did not reduce but increased luciferase expression (Fig. 1A) inferring the assay predominantly measured BACE1-mediated amyloidogenic processing.

To increase the dynamic range of the APP processing-dependent luminescence, the adapter protein Fe65 was cotransfected and luciferase expression was measured 24 hours later. This enhanced luciferase expression approximately 10-fold (Fig. 1B) confirming that the assay was sensitive to physiological stabilizers of the AICD. The BACE1 sensitivity of the assay predicted that familial AD mutations known to enhance Aβ production, such as the Swedish mutation of APP (K595 N/M596 L) (Citron et al., 1992) would increase luciferase expression. Site directed mutagenesis of the APP-Gal4 vector to incorporate these point mutations resulted in a 2-fold increase in luciferase expression compared with non-mutated APP, whereas remaining sensitive to DAPT (Fig. 1C). Taken together, these data confirmed the assay tractability and the resultant activity shifts were consistent with the assay being predominantly a reporter of amyloidogenic APP processing.

### 3.2 Flavonoid screen identified inhibitors of APP-Gal4 dependent luciferase expression

To date, no comparative systematic study of flavonoid bioactivity at a physiologically relevant concentration has been conducted. To address this, a library of flavonoids were selected and screened for modulatory effects on the APP-Gal4 driven luciferase assay. The purpose of the screen was to perform basic structure-activity analysis to assign favorable inhibitory activity at APP processing to defined subfamilies of flavonoids. Flavonoids were tested at 100 nM as a physiologically relevant concentration. Four flavonoids inhibited APP-Gal4 dependent luciferase expression at 100 nM (Table 1): epigallocatechin (57%), fisetin (67%), pelargonidin chloride (61%), and sinensetin (51%).

**Table 1:** Flavonoid screen identified inhibitors of APP-Gal4 dependent-luciferase expression

Flavonoid group	Compound name	Activity compared to control in % 100 nM
Flavonol	Fisetin^a^	67.7 ± 11.7
	Kaempferol	103 ± 54.1
	Kaempferol 3O rutinoside	83.0 ± 44.7
	Quercetin	98.6 ± 18.1
Flavone	Apigenin	90.9 ± 27.9
	Apigenin 7O glucoside	102 ± 13.6
	Coumarin	137 ± 15.1
	Diosmetin	90.4 ± 14.3
	Hyperoside	94.1 ± 14.3
	Sinensetin^b^	51.1 ± 26.1*
Flavanone	Hesperetin	134 ± 14.5
	Narirutin	126 ± 20.7
Anthocyanin	Cyanidin chloride	111 ± 39.8
	Delphinidin chloride	114 ± 32.9
	Pelargonidin chloride^a^	61.4 ± 6.23
Flavanol	(+) Catechin	97.6 ± 14.7
	(−)-Epicatechin	107 ± 35.1
	Epicatechin gallate	115 ± 28.1
	Epigallocatechin^b^	57.2 ± 23.3*
	Epigallocatechin gallate	91.9 ± 33.6

Five DIV primary cortical neurons were cotransfected with 0.5 μg pRC-APP-Gal4, pFR-luciferase, and pRL-TK-Renilla, after 0.5 hours; cells were treated with 100 nM flavonoid compound for 24 hours. Cells were lysed and levels of luminescence quantified using the Dual-Glo luciferase kit according to the manufacturer's instructions.

Key: ANOVA, analysis of variance; SD, standard deviation.

a Flavonoids showing largest inhibition of luciferase expression.

b Flavonoids showing largest inhibition of luciferase expression and caused significant reduction in luciferase expression (* *p* = 0.05, 1-way ANOVA with Bonferroni posttest). Values expressed as mean ± SD (n = 4).

### 3.3 (−)-Epicatechin and epigallocatechin identified as potent inhibitors of APP processing

**Fig. 2:**
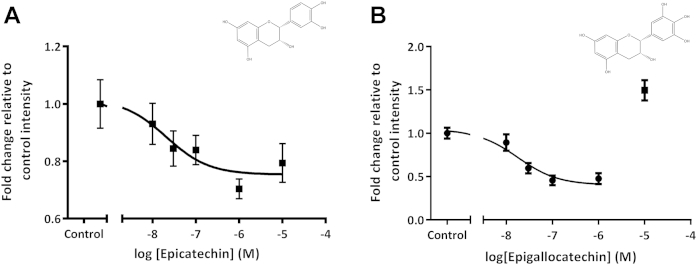
(−)-Epicatechin and epigallocatechin are potent inhibitors of APP processing. (A) (−)-Epicatechin is a 3-ringed polyphenol with hydroxyl substitutions at 7 of the A, 3 of C ring, and 4′, 5′ of the B ring. Wild-type primary neuronal cultures were transfected (see Section 2) 0.5 hours later treated with (−)-epicatechin for 6 hours. Treatment inhibited βγ-secretase-dependent APP processing with an EC_50_ of 20.5 nM. (B) Epigallocatechin has the same core structure as (−)-epicatechin with additional hydroxyl substitution at position 3′ of the B ring. Wild-type primary neuronal cultures were transfected (see Section 2) 0.5 h later treated with epigallocatechin for 24 hours. Epigallocatechin potently inhibited APP processing with EC_50_ of 18.6 nM. Abbreviation: APP, amyloid precursor protein.

The bioavailability and metabolism of flavanols have been studied in some depth (Abd El Mohsen et al., 2002, Wang et al., 2012) and oral administrations of different members of this family have reported bioactivity in rodent models (van Praag et al., 2007) and in humans (Schroeter et al., 2006). Therefore, because of this established bioavailability, flavanols were the focus of more detailed concentration and kinetic analyses. Neurons were treated with (−)-epicatechin, epicatechin gallate, epigallocatechin, or epigallocatechin gallate (0.01–10 μM) for 6 and 24 hours. Single administrations of flavanols in humans are quickly absorbed and broken down, with levels back to baseline after 12 hours (Renouf et al., 2013), therefore a more acute, 6 hour treatment was performed. (−)-Epicatechin potently reduced luciferase expression (EC_50_ of 20.5 nM) at 6 hours (Fig. 2A) but not 24 hours (data not shown). In contrast, epigallocatechin potently reduced luciferase expression (EC_50_ of 18.6 nM) at 24 hours (Fig. 2B) but not 6 hours (data not shown). Epicatechin gallate and epigallocatechin gallate showed no activity at 6 hours or 24 hours (data not shown). The effects of (−)-epicatechin and epigallocatechin were also concentration dependent, showing biphasic activity, no longer inhibiting APP processing at 10 μM. Epigallocatechin (24 hours) significantly potentiated luciferase expression at 10 μM, suggesting activity at a secondary cellular target at this higher concentration.

### 3.4 (−)-Epicatechin reduced Aβ production in TASTPM primary neurons

**Fig. 3:**
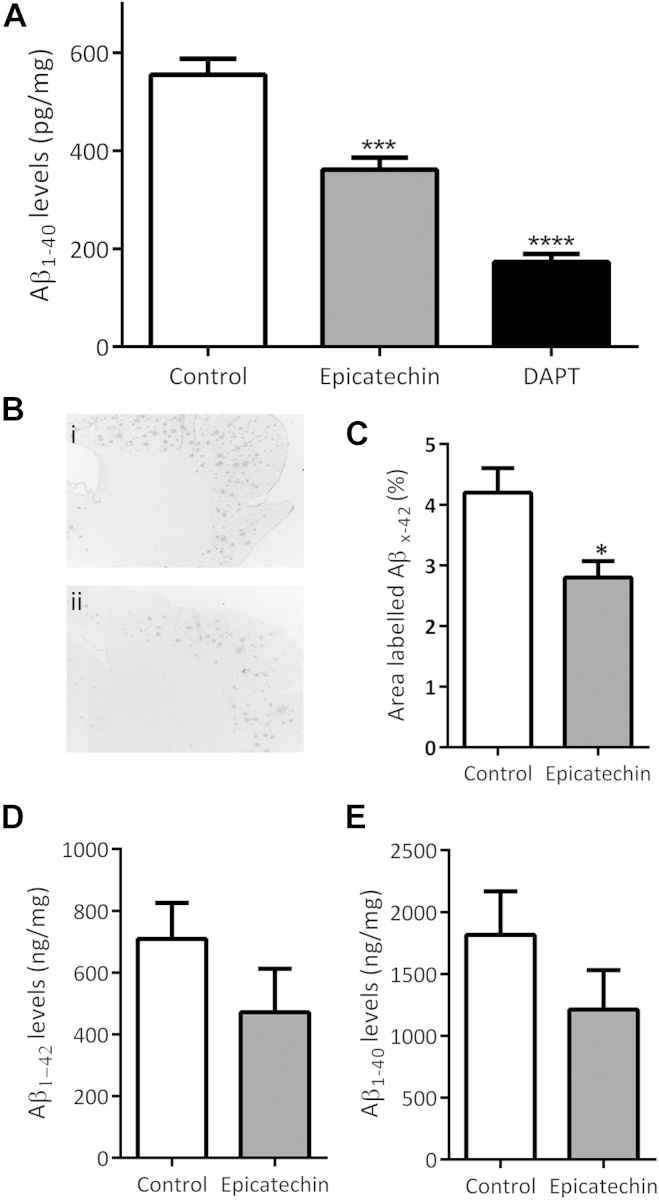
(−)-Epicatechin reduces Aβ levels in TASTPM transgenic mouse model. (A) Seven DIV TASTPM primary cortical neurons were treated for 6 hours with either vehicle, 100 nM (−)-epicatechin or 10 μM DAPT. Aβ_1-40_ levels were assessed by ELISA using monoclonal 1A10. *** *p* < 0.001 and **** *p* < 0.0001 1-way ANOVA with Bonferroni posttest, n = 6. (B) A 21-day oral delivery of (−)-epicatechin (15 mg/d in drinking water) reduced Aβ plaque load in TASTPM transgenic mouse. Representative 5 μm sagittal section taken after completion of feeding study. Mouse monoclonal Aβ_x-42_ specific 20G10 used to stain (i) control and (ii) (−)-epicatechin (15 mg/d) treated TASTPM mice. Scale bar = 200 μM. (C) Quantified as percentage area labeled with Aβ_x-42_ antibody. * *p* < 0.05 Student t test n = 5. (D and E) Trend toward decreasing levels of soluble Aβ_1-42_ and Aβ_1-40_ levels following (−)-epicatechin diet, n = 4. Abbreviations: Aβ, amyloid beta; ANOVA, analysis of variance; ELISA, enzyme-linked immunosorbent assay.

Despite the superior efficacy of epigallocatechin, the fact that it enhanced APP-cleavage dependent luciferase expression at 10 μM suggested increased amyloidogenic processing at high concentrations making it unsuitable for consideration as a potential intervention for AD. (−)-Epicatechin, although as potent as epigallocatechin, showed no promotion of luciferase expression at 10 μM and is blood brain barrier permeable (Abd El Mohsen et al., 2002, Wang et al., 2012). (−)-Epicatechin has also been shown to have positive effects on memory in in vivo rodent models (van Praag et al., 2007). For these reasons, (−)-epicatechin was taken forward for further investigation. TASTPM mice express the APP K595 N/M596 L Swedish double point mutation and the PS1 M146 V familial mutation. They develop extensive amyloid β pathology as a result of enhanced amyloidogenic processing from 3 months and exhibit cognitive deficits evident from 6 months of age (Howlett et al., 2004). Therefore, an in vitro assessment of (−)-epicatechin bioactivity was undertaken in primary neurons derived from TASTPM embryos. Neurons were treated with (−)-epicatechin (100 nM for 6 hours), and the levels of Aβ released into the media were assessed by ELISA. The γ-secretase inhibitor DAPT led to a 3-fold reduction in Aβ_1-40_ levels indicating that the Aβ detected in the media resulted from γ-secretase mediated APP cleavage events and not from cytotoxicity or nonspecific proteolysis (Fig. 3A). (−)-Epicatechin decreased Aβ_1-40_ levels by approximately 30%, consistent with inhibition of βγ processing of APP as suggested by the initial in vitro work (Fig. 3A). Aβ_1-42_ levels were below the detection limit of the assay (data not shown).

### 3.5 Oral (−)-epicatechin delivery reduces Aβ pathology in TASTPM mice

To explore the potential of (−)-epicatechin as an effective protective molecule against AD-like pathology, 7-month-old TASTPM mice were given 15 mg/d (−)-epicatechin in drinking water for 21 days. Animals were then sacrificed and cortical Aβ_x-40,42_ plaque load and soluble Aβ levels were assessed. Aβ plaque load was reduced by approximately one-third (Fig. 3B and C), whereas levels of soluble Aβ_1-40_ and Aβ_1-42_ both showed downward trends (Fig. 3D and E). These observations show, for the first time, that short term administration of orally delivered (−)-epicatechin reduced Aβ pathology in the cortex of an aged transgenic model of AD.

For the development of (−)-epicatechin as an AD treatment or to be able to make dietary recommendations it is important to address the mechanism of action. Flavanols have been shown previously to reduce Aβ pathology (Rezai-Zadeh et al., 2008, Wang et al., 2008), however the mechanism remains controversial.

### 3.6 (−)-Epicatechin treatment does not affect APP695, sAPPα, or α-CTF levels but reduces β-CTF formation

**Fig. 4:**
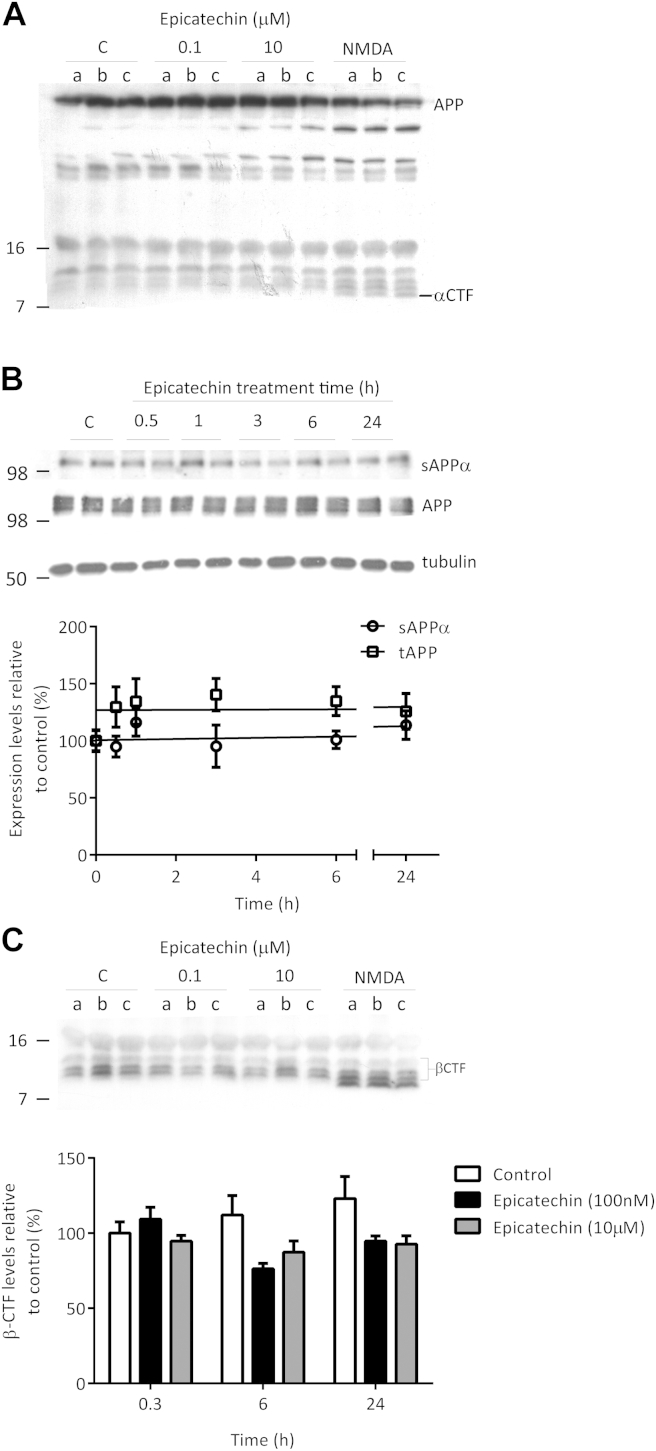
(−)-Epicatechin causes a trend toward reduced β-CTF without affecting APP levels or α-secretase mediated APP metabolites. (A) (−)-Epicatechin does not cause a time or concentration dependent alteration in total APP levels or α-CTF levels as seen with 50 μM NMDA (a) 20 minutes, (b) 6 hours, and (c) 24 hours treatment. Western blot was performed with monoclonal CT20 (1:75,000) antibody. (B) No significant change in sAPPα or total APP695 protein levels following 0.1 μM (−)-epicatechin over time course from 0.5 to 24 hours. Western blot analysis was performed with monoclonal antibody supernatant 5G11 for sAPPα (1:9), antibody 22C11 for APP (1:5000), and T8328 β tubulin (1:10,000), representative blots are shown. (C) (−)-Epicatechin (0.1 μM) caused a trend toward a time-dependent reduction in β-CTF levels in the absence of increased α-CTF levels measured at (a) 20 minutes, (b) 6 hours, and (c) 24 hours treatment, n = 3. Abbreviation: APP, amyloid precursor protein; CTF, C-terminal fragments.

Although these results were consistent with an inhibition of βγ-processing there were a number of potential mechanisms which could explain this phenomenon as reduction in Aβ levels could result from: inhibition of BACE1 or γ-secretase activity, increased α-secretase activity, or following changes in APP expression. To investigate if the inhibitory actions of (−)-epicatechin were the result of changes in APP expression, primary neurons were treated with 100 nM (−)-epicatechin over 24 hours, and APP695 levels in total cell lysates were measured by Western blot analysis using either a C-terminal (Fig. 4A) or an N-terminal (Fig. 4B) directed APP antibody. No significant change in total APP levels were observed (Fig. 4A and B), indicating that (−)-epicatechin was not acting to downregulate APP levels either through inhibition of translation or increased protein turnover. Previous studies investigating the effects of structurally similar flavanols on APP processing have suggested modulation of α-secretase as the likely mechanism of action (Fernandez et al., 2010, Obregon et al., 2006). To investigate this possibility, 6 DIV primary neuronal cultures were treated with (−)-epicatechin (0.1 or 10 μM) or a known stimulator of α-secretase, NMDA, for 20 minutes, 6 hours and 24 hours, and the production of APP CTFs was measured. NMDA (50 μM) caused a time-dependent increase in the levels of α-CTF (Fig. 4A) consistent with enhancement of α-secretase processing as previously reported (Hoey et al., 2009). In contrast, (−)-epicatechin showed no time or concentration-dependent changes in α-CTF levels suggesting that α-secretase was unlikely to be the primary target (Fig. 4A). This was further supported by analysis of sAPPα secretion which was not altered following treatment with (−)-epicatechin. As α-secretase was not affected by (−)-epicatechin treatment the effect on β-CTF formation was examined more closely. As before, neuronal cultures were treated with (−)-epicatechin (0.1 or 10 μM) or NMDA (50 μM), for 20 minutes, 6 hours and 24 hours, and the production of APP CTFs was measured. (−)-Epicatechin treatment resulted in a clear trend toward reduced levels of β-CTF fragments likely representing lower levels of C99 (b-CTF), p-β′-CTF (C89) and/or β′-CTF (Fig. 4C), and consistent with reduced β-secretase processing.

### 3.7 (−)-Epicatechin modulates BACE1 enzyme activity indirectly

**Fig. 5:**
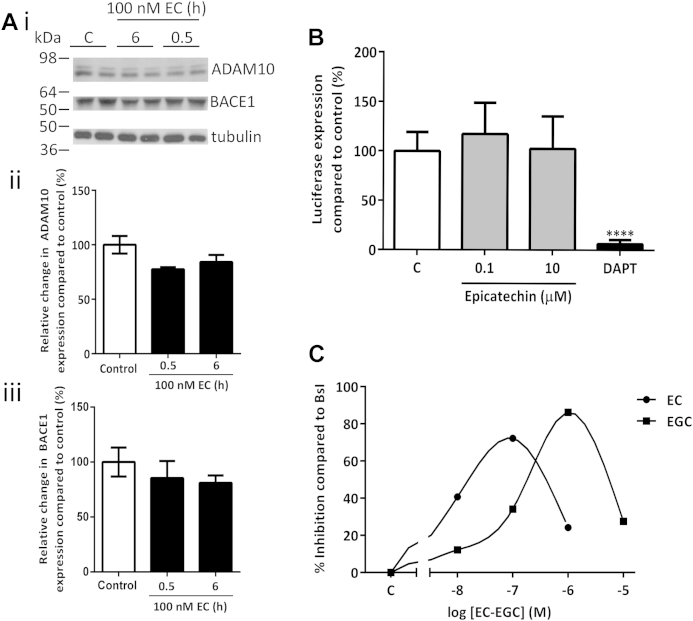
(−)-Epicatechin indirectly modulates BACE1 activity independent of BACE1 or ADAM10 expression changes and without affecting notch activity. (A) Epicatechin treatment does not affect α- or β-secretase levels in primary cortical neurons as seen following vehicle (C) or (−)-epicatechin (0.1 μM) for 30 minutes or 6 hours. Western blot analysis was performed for ADAM10 (735–749) (1:2000), BACE1 (2C13) (1:2000), or β tubulin (1:10,000). (i) Representative blots, (ii) ADAM10 quantification, and (iii) BACE1 quantification. N = 4 from 2 independent experiments. (B) (−)-Epicatechin does not affect γ-secretase activity: 5 DIV WT primary neuronal cultures were transfected with 0.5 μg Notch-Gal4, pFR-luciferase, and pRL-TK-Renilla and treated with (−)-epicatechin or 10 μM DAPT 0.5 hours later for 6 hours. **** *p* < 0.0001 1-way ANOVA with Bonferroni posttest. (C) Reduction of BACE1 activity in primary cell lysates treated with (−)-epicatechin and epigallocatechin. Primary neuronal cultures were treated with (−)-epicatechin (6 hours) or epigallocatechin (24 hours), the cells were then lysed and levels of BACE1 activity was measured using a synthetic peptide substrate. Data are represented as percentage inhibition compared with βsI. Abbreviations: ANOVA, analysis of variance; βsI, β-secretase inhibitor; WT, wild type.

Alterations in APP processing could quite possibly result from changes in the expression of the secretases involved. To investigate, these neuronal cultures were treated with (−)-epicatechin (100 nM) for 0.5–6 hours, and levels of the primary α-secretase ADAM10 and BACE1 were measured by immunoblotting. The levels of ADAM10 and BACE1 were not altered by (−)-epicatechin treatment (Fig. 5A), suggesting that the acute inhibitory effects on APP processing were not because of changes in secretase expression. An alternative hypothesis was that (−)-epicatechin was acting to inhibit to γ-secretase activity. To investigate whether (−)-epicatechin was acting through γ-secretase, a Notch-Gal4 luciferase reporter assay was used. Primary neuronal cultures were treated with (−)-epicatechin (0.1–10 μM) or DAPT (10 μM) for 6 hours and luciferase expression measured. DAPT caused a substantial reduction (approximately 90%) in luciferase expression demonstrating that the assay was sensitive to γ-secretase cleavage of Notch. (−)-Epicatechin did not affect luciferase expression at either concentration tested (Fig. 5B), indicating its bioactivity was not at the level of γ-secretase. As α- and γ-secretases did not appear to mediate the inhibitory actions of (−)-epicatechin, and there were no changes in secretase expression; activity at BACE1 was considered the most likely target. To address this, a cell free recombinant BACE1 assay was used. (−)-Epicatechin (0.01–10 μM) had no direct effect on BACE1 activity in this assay suggesting it was not acting as a direct catalytic inhibitor (data not shown). Alternatively, noncatalytic actions might be responsible for the inhibitory effects of (−)-epicatechin. Cultured neurons were therefore, treated with (−)-epicatechin (0.01–10 μM) for 6 hours, lysates prepared and incubated with a synthetic BACE1 substrate to measure endogenous BACE1 activity. Under these conditions (−)-epicatechin (100 nM) reduced BACE1 activity by 72% compared with the BACE inhibitor βsI (Fig. 5C). In comparison, epigallocatechin caused maximum inhibition (86%) at 1 μM. At higher concentrations of (−)-epicatechin and epigallocatechin there was no inhibition of BACE1 activity.

## 4 Discussion

The current failure rate of AD drugs has driven research interests toward alternative small molecules with therapeutic potential for reducing risk or slowing progression of dementia. Mounting evidence suggests that selected dietary flavonoids, polyphenolic compounds with steroid-like structures, are able to reduce Aβ pathology and show positive effects on learning and memory (Williams and Spencer, 2012). There is however, a lack of consensus on the precise identities of the bioactive molecules and mechanisms underlying these potentially beneficial effects as the concentrations used in many studies far exceed those achievable in vivo and flavonoids generally have very poor bioavailability.

In this study, an in vitro screen of dietary flavonoids in primary neurons led to the identification of (−)-epicatechin and epigallocatechin as potent (nanomolar) inhibitors of amyloidogenic APP processing. Studies in aged TASTPM transgenic mice showed that oral administration of (−)-epicatechin reduced Aβ pathology. This reduction was seen following 21 days of (−)-epicatechin treatment, the first time oral administration has been shown to be effective on such a short timescale. Mechanistic studies revealed the likely mode of action of (−)-epicatechin was through indirect, noncatalytic BACE1 inhibition and not through modulation of either α-secretase or γ-secretase activity.

The initial unbiased in vitro flavonoid screen was conducted using an APP-Gal4-driven luciferase gene reporter assay which has been shown to preferentially report amyloidogenic processing when used in primary cultured neurons (Hoey et al., 2009, Hoey et al., 2013). This was confirmed in this study as luciferase gene reporter expression was inhibited by commercial β- and γ- secretase inhibitors, enhanced by Fe65 cotransfection and increased following the introduction of APP mutations known to favor Aβ formation. Inhibition of α-secretase activity tends to enhance luciferase expression. This assay is therefore, a powerful technique for screening compounds with potential bioactivity at different points in the pathways regulating APP processing and is not simply a tool for identifying direct catalytic inhibitors of β- and γ- secretase. Using this approach, 4 flavonoids were identified that reduced APP cleavage-dependent luciferase expression at 100 nM (24 hours): fisetin, pelargonidin, sinensetin, and epigallocatechin. Fisetin has previously been identified as an activator of signaling pathways implicated in learning and memory (Maher et al., 2006). Pelargonidin as the major constituent of strawberries has been implicated in reversing age-related cognitive decline (Joseph et al., 1999). Sinensetin has been much less studied but very recently was shown to activate cyclic AMP response element-mediated transcription in rat hippocampal neurons, a key pathway in neuroprotection (Kawahata et al., 2013) and to have anti-angiogenic effects in a zebrafish model (Lam et al., 2012). Whether these activities of fisetin, pelargonidin, and sinensetin are related to or additional to, the inhibitory actions at APP processing is unknown. Epigallocatechin was perhaps the most significant positive hit from the assay, as it is a member of an intensively studied family of flavanol molecules called the catechins which have known bioavailability in a variety of mammalian models (Ho et al., 2013, van Praag et al., 2007) and have been previously postulated to have therapeutic potential for neurodegeneration (Mandel et al., 2011, Pasinetti, 2012).

Further kinetic analysis of the catechin family revealed that (−)-epicatechin, in addition to epigallocatechin, possessed potent inhibitory actions but only when applied for shorter time points, and this inhibition was not apparent at longer time points potentially because of metabolism into an inactive form or differences in membrane permeability. Indeed, (−)-epicatechin was effective at 6 hours although epigallocatechin required 24 hours to reduce APP processing. This difference in biokinetics between (−)-epicatechin and epigallocatechin might be because of faster metabolism for (−)-epicatechin together with lesser membrane permeability for epigallocatechin. Concentration analyses revealed that (−)-epicatechin and epigallocatechin showed biphasic effects, losing their inhibitory properties and in the case of epigallocatechin, stimulating APP processing at higher micromolar concentrations. This biphasic profile has been reported previously for flavonoid modulation of the ERK and Akt signaling pathways in neurons (Vauzour et al., 2007) and suggests that concentration is a critical determinant of flavonoid selectivity.

(−)-Epicatechin and other monomeric proanthocyanidins have been shown to reach concentrations of 200–400 nM in rodent brain following oral dosage (Abd El Mohsen et al., 2002, Wang et al., 2012) and to promote pathways associated with learning and memory (Haque et al., 2008, van Praag et al., 2007, Wang et al., 2012) supporting the basic notion that oral administration of (−)-epicatechin might impact on AD pathology. Indeed, oral administration of (−)-epicatechin (approximately 15 mg/d) via drinking water for 21 days reduced Aβ pathology in TASTPM mice at an age when plaque burden was already well established (Howlett et al., 2004). This may go part way to explain the relatively modest reductions in pathology compared with other in vivo flavonoid studies such as those testing phenolic compounds, grape polyphenols and EGCG in Tg2576 mice, where administration was initiated at 5, 7, and 8 months respectively when plaque burden was not established until 9 months (Hamaguchi et al., 2009, Hsiao et al., 1996, Rezai-Zadeh et al., 2008, Wang et al., 2012). The reductions reported here are important however, as previously; only intraperitoneal injected flavonoid has been shown to have such short-term effects with a-7 day treatment of curcumin reducing plaques and Aβ levels (Garcia-Alloza et al., 2007). The precise mechanism underlying this favorable reduction in Aβ pathology is as yet unclear and serious consideration needs to be given as to whether inhibitory actions at BACE alone could account for such a dramatic reduction in Aβ pathology following only 21 days of administration. Flavanols have been proposed to have multimodal activities (Mandel et al., 2008), concurrently acting at multiple targets and could potentially impact on Aβ aggregation by favoring the formation of off-target oligomers (Ehrnhoefer et al., 2008) but only if micromolar concentrations could be achieved in vivo. This is not a mechanism that has been addressed here as the focus was on APP processing, but the ability of (−)-epicatechin to disrupt oligomeric Aβ formation should be tested under these conditions. Further studies should assess the effects of (−)-epicatechin on Aβ oligomer formation and clearance. Epigallocatechin-3-gallate (EGCG) has also been reported to increase α-secretase activity through increased maturation of ADAM10 (Fernandez et al., 2010, Rezai-Zadeh, 2005), but this does not appear to be the primary mechanism involved here as there was no evidence of a change in the levels of α-CTFs, sAPPα, or ADAM10 following (−)-epicatechin treatment. This difference could be because of cell type specificity, different modes of action because of lack of the gallic acid moiety in (−)-epicatechin or simply because of differences in the concentrations used, and it is quite possible that actions at α-secretase will be observed under different dosing regimes. The most direct potential mechanism for the observed (−)-epicatechin effect would be inhibition of BACE1, and a number of studies have suggested this as a potential mode of action for flavonoids. *In silico* docking studies suggested a number of flavonoids might act as direct catalytic inhibitors of BACE1 (Shimmyo et al., 2008). (−)-Epicatechin and epigallocatechin did not inhibit BACE1 activity in a recombinant enzyme assay, suggesting this mechanism of action is unlikely. Treatment with (−)-epicatechin and epigallocatechin did, however, reduce endogenous BACE1 activity suggesting an indirect inhibitory mechanism of action. How this is achieved is unclear but could involve actions at an allosteric site, posttranslational modification, downregulation of BACE1 expression, or modulation of BACE1 localization as recently reported with the plant-derived phytosterol stigmasterol (Burg et al., 2013). With respect to the development of (−)-epicatechin for use in humans an important consideration was the potential for inhibitory actions at γ-secretase as there have been a number of recent clinical trial failures for AD drugs because of off target effects at notch, leading to gastrointestinal and immune cell toxicity (Doody et al., 2013, Imbimbo et al., 2011). (−)-Epicatechin did not inhibit notch cleavage at any concentration tested, suggesting that this is unlikely to be a significant hurdle to the development of (−)-epicatechin as a potential treatment or prophylactic for AD. Before that can happen the bioavailability and pharmacokinetics of (−)-epicatechin and its principle in vivo metabolites will need to be thoroughly addressed, and the loss of favorable flavanol activity at high concentrations could be an issue.

In conclusion, this study has identified a single flavanol, (−)-epicatechin, to be effective at reducing Aβ production and pathology in wild-type neurons and in a transgenic model of AD and that this is most likely through modulation of BACE1 activity. Given that Aβ toxicity is almost certainly initiated at presymptomatic stages of AD, any potential benefit from an (−)-epicatechin intervention would be most likely achieved through a risk reduction strategy rather than as a treatment. The challenge now is to move beyond the epidemiology which has hinted at positive effects of flavonoid rich diets on the development of dementia (Dai et al., 2006, Kuriyama et al., 2006, Letenneur et al., 2007), into clinical trials to directly test efficacy in at risk individuals or those with mild cognitive impairment.

## Disclosure statement

Jill C. Richardson is an employee of GlaxoSmithKline, and David R. Howlett was an employee of GlaxoSmithKline when the work was undertaken, Michael S. Perkinton is an employee of Astra Zeneca Ltd. Other than employment and shareholding in GlaxoSmithKline, the authors declare no conflicts of interest.
